# A Case of Horner's Syndrome following Ultrasound-Guided Infraclavicular Brachial Plexus Block

**DOI:** 10.1155/2012/125346

**Published:** 2012-08-22

**Authors:** Trabelsi Walid, Belhaj Amor Mondher, Lebbi Mohamed Anis, Ferjani Mustapha

**Affiliations:** Departement of Anesthesia and Intensive Care Unit, Tunisian Military Hospital, 1002 Tunis, Tunisia

## Abstract

Horner's syndrome results from paralysis of the ipsilateral sympathetic cervical chain (stellate ganglion) caused by surgery, drugs (mainly high concentrations of local anesthetics), local compression (hematoma or tumor), or inadequate perioperative positioning of the patient. It occurs in 100% of the patients with an interscalene block of the brachial plexus and can also occur in patients with other types of supraclavicular blocks.In this case report, we presented a case of Horner's syndrome after performing an ultrasound-guided infraclavicular brachial plexus block with 15 mL of bupivacaine 0.5%. It appeared 40 minutes after the block with specific triad (ptosis, miosis, and exophtalmia) and quickly disappears within 2 hours and a half without any sequelae. Horner's syndrome may be described as an unpleasant side effect because it has no clinical consequences in itself. For this reason anesthesiologists should be aware of this syndrome, and if it occurs patients should be reassured and monitored closely.

## 1. Introduction

Horner's syndrome may correspond to a diffusion of local anesthetics in prevertebral spaces ultimately involving the sympathetic nerves and communicating with cervical nerve trunks [1]. It results from paralysis of the ipsilateral sympathetic cervical chain (stellate ganglion) caused by surgery, drugs (mainly high concentrations of local anesthetics), local compression (hematoma or tumor), or inadequate perioperative positioning of the patient [2, 3].

Interscalene and supraclavicular blocks of the brachial plexus are the main anesthetic techniques associated with this syndrome [4–6].

## 2. Case Report

After her written consent, a 52-year-old healthy woman (74 kg, 168 cm), classified as American Society of Anesthesiologists physical status I (with a medical history of subtotal thyroidectomy under cervicotomy), was scheduled for carpal tunnel syndroms surgery under tourniquet device. Perioperative anesthesia consisted of an ultrasound-guided infraclavicular brachial plexus block. No premedications were applied to the case. An intravenous cannula was inserted into the contralateral arm, and a continuous infusion (crystalloid solution) was started. For the whole procedure the patient was routinely monitored with electrocardiogram (ECG), noninvasive blood pressure (NIBP) measurement, and pulse oximetry (SpO2).

The patient was in supine position, with the head facing away from the side to be anesthetized, and the arm were adducted and flexed at the elbow and resting over the chest or upper abdomen. Following the positioning, the area on the infraclavicular region to be operated was disinfected. A 10- to 12-MHz linear probe (Logiq 7 GE Health care, USA) was placed in the deltopectoral groove. Under sterile conditions and after subcutaneous infiltration, a 22 G insulated needle (Echoplex D 50 mm, Vygon, France) was inserted and advanced using in-plane needle-probe alignment. Injection of local anesthetic selectively surrounded each sonographically imaged brachial plexus cord with approximately 5 mL of 0.5% bupivacaine.

Block onset time was 15 minutes. There was no change in vital parameters. 40 minutes after block performance (and 10 minutes after beginning of surgery) the Horner's syndrome was observed. The patient did not have any respiratory difficulty. Surgery was completed successfully without any complication. Horner's syndrome vanished approximately two hours and a half later.

## 3. Discussion

This case report is the first in the literature to describe a Horner's syndrome after ultrasound-guided infraclavicular brachial plexus block.

Jandard et al. [7] reported a Horner's syndrome rate of 4%, Kilka et al. [8] 7%, Rettig et al. [9] 12%, and Neuburger et al. [10] 1%. There may be many causes of Horner's syndrome. It may be related to distribution of local anesthetic solution. There are different studies about distribution of local anesthetics and anatomy of the brachial plexus. Rodríguez et al. [11] found that local anesthetics, after both supraclavicular and interscalene blocks, were distributed above and below the clavicle, and conversely they found that after a coracoid block, local anesthetics remained below the clavicle in all patients.

Winnie [12] suggests that the brachial plexus is completely encased in a continuous tubular sheath from the interscalene groove to the axilla. On the other hand Beck et al. [13] showed, in an anatomical study, that connective tissue thickening could divide the neurovascular space of the brachial plexus into two compartments, possibly explaining the unidirectional spreading from the supraclavicular space to the infraclavicular space.

Horner's syndrome can be observed during continuous infusion or with a single large volume. Salengros et al. [14] observed delayed Horner's syndrome in a case during a continuous infusion through a catheter with infraclavicular block approximately 50 hours after the initial block. But Ilfeld et al. [15] published a series that examined patients managed with continuous postoperative infraclavicular local anesthetic infusion. They did not report any signs of Horner's syndrome.

Causes of Horner's syndrome are large accumulation of local anesthetic solution or the atypical proximal migration of the solution above the clavicle toward the supraclavicular paravertebral area. Sometimes Horner's syndrome can be observed without catheter placement, above 40 mL volumes [7, 8]. In our case, we think that history of cervicotomy may be responsible for this atypical syndrome caused by anatomical changes in local anesthetic diffusion space, not its volume.

When the rates of complications between the supraclavicular and infraclavicular approaches are compared impairment in diaphragmatic movements can apparently be rated as 100% for interscalene, 50% to 77% for supraclavicular, 24% to 26% for proximal infraclavicular and 0% for more distal infraclavicular blocks [4, 6, 9, 11]. According to Rettig et al. [9], Horner's syndrome is a clinically significant sign (100%) that predicts changes in hemidiaphragmatic movement; however, in their patients, changes in hemidiaphragmatic movement weres also observed without Horner's syndrome. They suggested that the phrenic nerve and sympathetic trunk are more separated. We did not observe altered respiratory functions or dysphonia in our patient.

Although Horner's syndrome had no clinical consequences to this patient, it may be described as an unpleasant side effect. It has the potential to lead to patient anxiety, discomfort, and dissatisfaction. For this reason anesthesiologists should be aware of this syndrome, and if it occurs, patients should be reassured and monitored closely.
